# Infectivity Upsurge by COVID-19 Viral Variants in Japan: Evidence from Deep Learning Modeling

**DOI:** 10.3390/ijerph18157799

**Published:** 2021-07-22

**Authors:** Essam A. Rashed, Akimasa Hirata

**Affiliations:** 1 Department of Electrical and Mechanical Engineering, Nagoya Institute of Technology, Nagoya 466-8555, Japan; ahirata@nitech.ac.jp; 2 Department of Mathematics, Faculty of Science, Suez Canal University, Ismailia 41522, Egypt; 3 Center of Biomedical Physics and Information Technology, Nagoya Institute of Technology, Nagoya 466-8555, Japan

**Keywords:** COVID-19, forecasting, deep learning, viral variants

## Abstract

The significant health and economic effects of COVID-19 emphasize the requirement for reliable forecasting models to avoid the sudden collapse of healthcare facilities with overloaded hospitals. Several forecasting models have been developed based on the data acquired within the early stages of the virus spread. However, with the recent emergence of new virus variants, it is unclear how the new strains could influence the efficiency of forecasting using models adopted using earlier data. In this study, we analyzed daily positive cases (DPC) data using a machine learning model to understand the effect of new viral variants on morbidity rates. A deep learning model that considers several environmental and mobility factors was used to forecast DPC in six districts of Japan. From machine learning predictions with training data since the early days of COVID-19, high-quality estimation has been achieved for data obtained earlier than March 2021. However, a significant upsurge was observed in some districts after the discovery of the new COVID-19 variant B.1.1.7 (Alpha). An average increase of 20–40% in DPC was observed after the emergence of the Alpha variant and an increase of up to 20% has been recognized in the effective reproduction number. Approximately four weeks was needed for the machine learning model to adjust the forecasting error caused by the new variants. The comparison between machine-learning predictions and reported values demonstrated that the emergence of new virus variants should be considered within COVID-19 forecasting models. This study presents an easy yet efficient way to quantify the change caused by new viral variants with potential usefulness for global data analysis.

## 1. Introduction

The global challenge caused by the COVID-19 pandemic is unavoidable and there has been significant mortality and damage to the global economy [1]. While the situation is expected to recover with the development and administration of vaccines [2], many countries are concerned with limitations associated with the vaccination process (WHO, https://covid19.who.int/ (accessed on 17 June 2021)). It becomes more challenging to continue strong restrictions on public movement or nation-wide lockdown with the global economy collapse [3]. Several territories have considered public awareness by requesting voluntary actions to reduce the spread of the pandemic [4,5,6]. However, the development of these policies requires an efficient forecasting process to provide appropriate instructions and proper timing.

In epidemiology, mathematical modeling of the viral spread is commonly used to understand the current and future infection risks. The most used models are the susceptible, infected, and recovered (SIR) [7] and the susceptible, exposed, infected, and recovered (SEIR) models [8]. These compartmental models was used to demonstrate several pandemics earlier to COVID-19. Moreover, several attempts are considered modifications of conventional compartmental models for more general and efficient forecasting (e.g., [9,10]). A review of COVID-19 forecasting models is in [11]. In this review, it was shown that deep learning models can reach to human expert level but it requires a relatively large amount of training data.

Several models have been developed for the prediction of potential risk, such as infection rate increases, using different data forms [12,13,14,15,16,17,18]. With the emerging of new virus mutations [19], it has become unclear how such forecasting models designed using data obtained at the first generation of the virus spread can still be efficient to predict effects from emerging variants of the virus. The SARS-CoV-2 variant, B.1.1.7 lineage (a.k.a. 20B/501Y.V1 variant of concern (VOC) 202012/01) was first identified in the UK. Since then, many other cases have been reported in different regions. The speed of this spread was suggested to be faster than expected, although quantitative discussion is difficult because of the presence of many other co-factors. It has been reported that the new UK variant B.1.1.7 (referred as the Alpha variant hereafter) has a 43%–90% higher effective reproduction number [20,21]. This new variant has become common in Japan as of March 2021 and the first case was reported on 25 December 2020.

In previous studies, human mobility was suggested to be one of the key factors in characterizing the spread of the virus [22,23,24,25]. The mobility data was used as a surrogate of public activities and indication of social distancing, which is known as a dominant factor associated with COVID-19 infections. In addition, meteorological data have been suggested as additional factors that influence viral spread [26,27,28,29,30,31]. A recent systematic review suggested that, among meteorological factors, temperature and humidity were significantly correlated with COVID-19 morbidity [32]. In other studies, parameters related to policy, pollution levels, and wind speed were also included, which may also be considered as potential factors [33,34]. Our previous study suggested that some of these factors are confounding factors.

Based on the above findings, we demonstrated that a machine learning model based on long short-term memory (LSTM) that had only three parameters; that is, mobility at a central station in each district, ambient temperature, and humidity, was enough to estimate daily positives cases (DPC) in several urban areas in Japan. From one year of data from six districts, the average relative error was slightly improved by considering meteorological factors [35]. We investigated the effect of viral variants on the speed of the spread in different districts of Japan. The discussion was based on machine learning predictions that were developed in our previous study based on past data for one year. If our previous model works even after the emergence of these new variants, the model and parameters could be useful for future predictions. If the speed of the spread of the new variant is different, then further consideration is needed for future predictions.

## 2. Materials and Methods

### 2.1. Data Collection and Processing

**Figure 1:**
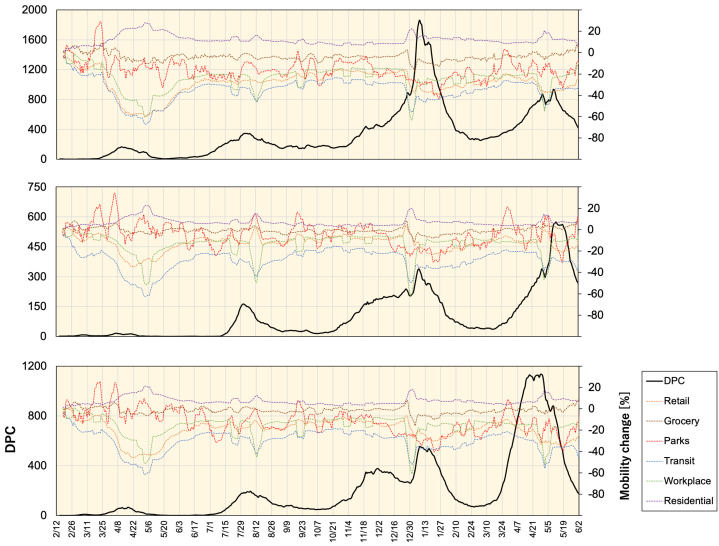
COVID-19 daily positive cases (DPC) and Google mobility change rates from baselines for Tokyo, Aichi, and Osaka (from top to bottom) from 19 February 2020 to 2 June 2021. Lines represent a 7-day average.

**Figure 2:**
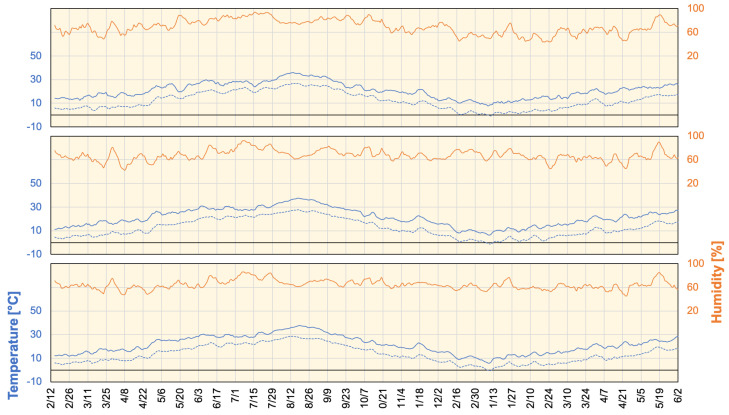
Maximum/Minimum daily temperature and average humidity for Tokyo, Nagoya, and Osaka (from top to bottom) from 19 February 2020 to 2 June 2021. Lines represent a 7-day average.

In this study, we considered data from six districts of Japan in which a remarkable number of SARS-CoV-2 variants were reported that resulted in the issuance of a national State of Emergency (SoE) during May–June 2021. The number of COVID-19 DPC were obtained from the online open data sources provided by the Japanese Ministry of Health, Labor, and Welfare (https://www.mhlw.go.jp/stf/covid-19/open-data.html (accessed on 28 May 2021)) and local district websites. Effective reproduction number (R) data were obtained from Toyo Keizai online resources (https://toyokeizai.net/sp/visual/tko/covid19/en.html (accessed on 15 June 2021)). The *R* value is computed using the following equation:

(1) $$R_{t}={\left(\frac{\sum_{i=1}^{s}DPC_{t-i}}{\sum_{i=s+1}^{2s}DPC_{t-i}}\right)}^{\mu /s},$$

where s=7 is the number of days for specific time period and μ=5 days is the mean generation time. Public movements were estimated from Google mobility reports (https://www.google.com/covid19/mobility/ (accessed on 21 May 2021)) that represented data global records from 15 February 2020. Google mobility reports showed the percentage of change in urban regions labeled as retail and recreation, grocery and pharmacy, parks, transit stations, workplaces, and residential in comparison with baseline data (median value from the 5-week period from 3 January, to 6 February 2020). Google mobility data, along with DPC in Tokyo, Aichi, and Osaka, are shown in Figure 1. Weather data measured at major cities within the target region were obtained from the Japan Meteorological Agency (https://www.jma.go.jp/jma/index.html (accessed on 28 May 2021)). Daily maximum/minimum temperature and average humidity that were acquired for Tokyo, Aichi, and Osaka are shown in Figure 2. Moreover, a reference representing the situation of working/vacation days is considered along with binary (1/0) labels representing national/local SoE call/release. All data were normalized to generate unified integrated training batches using the following equation.

(2) $${\tilde{y}}_{i}=\left(\beta -\alpha \right)\frac{y_{i}-\min\left(y\right)}{\max(y)-\min(y)}+\alpha ,$$

where α and β are scaling parameters, and *y* and $\tilde{y}$ are the originally acquired data and normalized values, respectively. The dataset described above was collected for Tokyo, Aichi, Osaka, Hyogo, Kyoto, and Fukuoka and was split into training/testing batches considering 15 different time periods as listed in Table 1, which demonstrated a stride of one week forward each. For each time period, all training data of the six districts were normalized and combined to generate more reliable training features in a single dataset.

**Figure 3:**
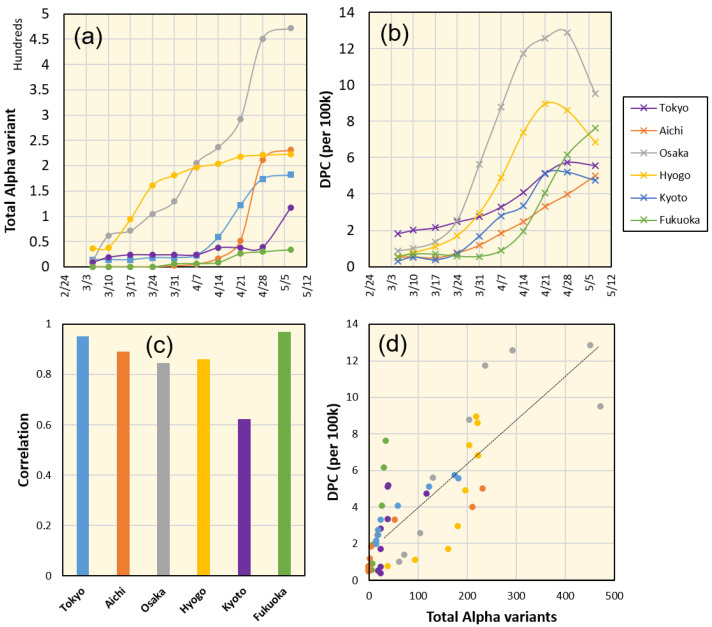
(**a**) Total confirmed cases with the Alpha variant and (**b**) DPC (per 100,000) in Tokyo, Aichi, Osaka, Hyogo, Kyoto, and Fukuoka. (**c**) Correlation between the total Alpha variant and the DPC per 100 k demonstrated high values. (**d**) Demonstration of time-independent high correlation between the Alpha variant and DPC ($R^{2}$ = 0.65).

The number of cases in which the viral variant was confirmed was acquired from the MHLW data port that was recently released (https://www.mhlw.go.jp/stf/seisakunitsuite/newpage_00054.html (in Japanese, accessed on 11 May 2021)). A sample of Alpha variant data is shown in Figure 3. The correlation between the changes in reported Alpha variant cases (confirmed by genome analysis) and DPC (scaled over 100,000 persons) in March/April 2021 is shown in Figure 3c,d. A high correlation was clearly demonstrated. However, as the data record of new viral variants is limited, we would like to further investigate this observation using a deep learning model trained with long-term data and validate the results obtained in several time frames. The effectiveness of this approach can be found in our previous study [35].

### 2.2. Forecasting Deep Learning Model

A deep LSTM neural network was used to estimate the number of DPC from a blend of different data obtained earlier. LSTM is known to perform efficiently in time-series data forecasting and regression. In our earlier study, we proposed a multi-path LSTM neural network that could successfully estimate the number of DPC given the data of different districts in Japan [35]. The results demonstrated remarkable forecasting with good accuracy. However, with the emergence of new viral variants, the effective reproduction number has been reported to be higher [20,21] and, therefore, the pattern of future data is expected to lose consistency with the earlier data that was used for training.

**Figure 4:**
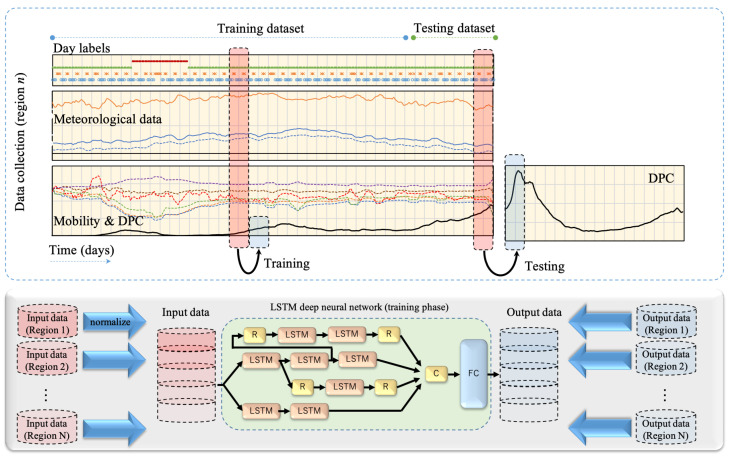
LSTM deep neural network is trained using day labels (working/vacation and normal/SoE), meteorological data (max/min temperature and average humidity), community mobility, and DPC. Network output is the estimated DPC. R, C, and FC indicate sequence reverse, concatenation, and fully connected layers. Training data acquired for different districts were normalized and merged for an efficient training process.

In this study, we set the time frame for input and output data to 14 days. In other words, the network was trained to estimate the DPC for the upcoming 14 days given the data measured in the earlier 14 days, as shown in Figure 4. Moreover, we also included the public mobility measure with a wider scope by including all spots covered by the Google mobility reports, while in our earlier study [35], we considered mobility around major transport stations only. More detailed mobility data is expected to improve the model accuracy by learning the contribution of different urban regions on COVID-19 morbidity. We also considered including binary labels to demonstrate the working day status and call of SoE. This was based on the observation that the DPC were influenced by the weekday status and SoE. The fully connected (FC) layer was set to the four levels; that is, 3k, 3k, 1.5k, and 150, of neurons and the output layer had 14 neurons (i.e., number of estimated days). The network architecture shown in Figure 4 was implemented using Wolfram Mathematica (R) ver. 12.1 with LSTM cells (each output vector was 300 elements). The selection of network parameters was optimized as detailed in an ablation study in [35]. The software was deployed on a workstation with four Intel (R) Xeon CPUs (3.6 GHz), three NVIDIA GeForce 1080 GPUs, and 128 GB memory. Different training/testing data samples were used for a better understanding of the performance of the forecasting model in different phases of the viral variant spread. The network training was conducted with a batch size of 16 over 500 training epochs.

### 2.3. Validation Metrics

The relative error was used as a measure of estimation accuracy and was computed as follows:

(3) $$E_{i}=\frac{|y_{i}-{\hat{y}}_{i}|}{y_{i}},$$

where $y_{i}$ and ${\hat{y}}_{i}$ are the real and estimated DPC in day *i*.

## 3. Results

### 3.1. Selection of Data Blend

An initial study was conducted to evaluate different scenarios of input data to verify the most appropriate data blend. We consider four scenarios that consider mobility data exclusion (Scenario 1), meteorological data exclusion with transit mobility inclusion (scenario 2), meteorological data exclusion with all mobility inclusion (scenario 3), and all data inclusion as shown in Table 2. Data for training and testing are set to periods 12–15 in Table 2. Average error values of the four time periods for all study districts is shown in Table 2. The preliminary study indicate that inclusion of full mobility information with meteorological data (scenario 4) would likely be the optimal choice.

### 3.2. Prediction of DPC

**Figure 5:**
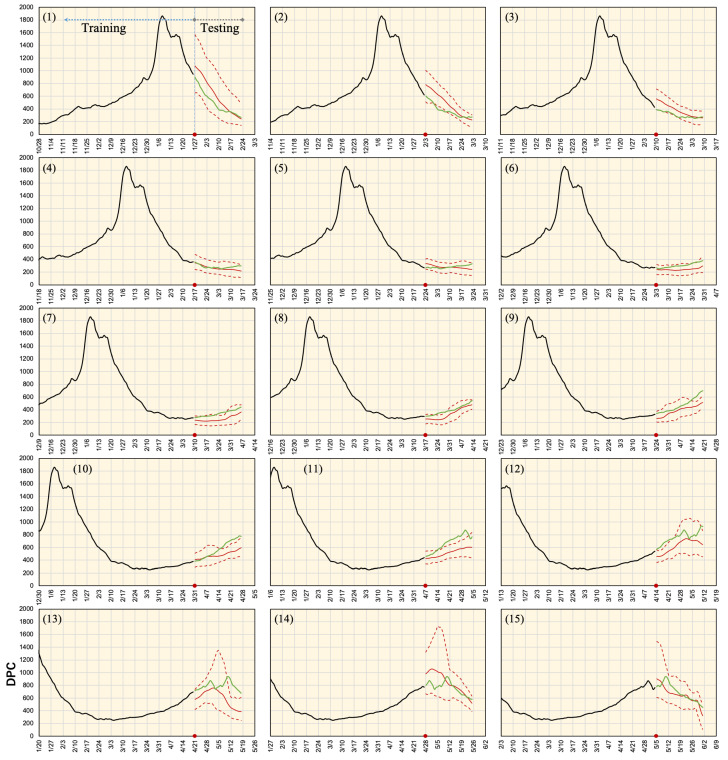
Actual and estimated DPC in Tokyo for different time phases as defined in Table 1. Black and green colors demonstrate actual reported data used for training and validation, respectively. Solid and dashed red lines are the average and maximum/minimum bounds, respectively. Earlier data records from 15 February 2020 are also included in the network training.

**Figure 6:**
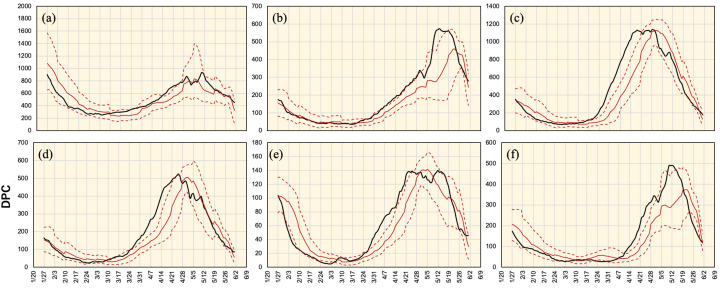
Actual (black) and average estimated (red) DPC in (**a**) Tokyo, (**b**) Aichi, (**c**) Osaka, (**d**) Hyogo, (**e**) Kyoto, and (**f**) Fukuoka within all 15 time periods. Dotted lines represent average maximum and minimum estimates.

The network was trained and tested using input data in different sets of time frames to validate the forecasting accuracy and network robustness. In each time frame, the testing data was validated with the stride of a single day. Different forecasting values were used to compute the maximum, minimum, and average estimates. Results obtained for Tokyo over the different 15 time periods are shown in Figure 5. The forecasting demonstrated different patterns in different time periods. Moreover, variations were relatively small in time periods 4–9. An average for data obtained from all time periods for the six districts is shown in Figure 6. In Tokyo, a high consistency was found between the estimated and observed values in almost all-time frames. The real values were always within the estimated range except for a single week (mid-April). In Aichi, good matching was observed between the estimated and observed values in the period earlier to mid-April with network underestimation on later days. Differences became significant in mid-May and accuracy was retrieved again in late-May. Osaka represents the extreme case where the estimated DPC were highly underestimated from mid-March to mid-April. During this period, true values were above the maximum forecasting boundary. The same pattern was observed in Hyogo, but with a smaller capacity. Kyoto demonstrated a mild mismatching between the network estimate and real values from late-March to late-April as the real data curve was above the maximum network estimate. Finally, Fukuoka data were underestimated from mid-April to mid-May and were overestimated later. In general, network estimations for the period before mid-March (and later than mid-May) had higher consistency with real values. In contrast, network forecasting for mid-March to mid-May had low accuracy. Quantitative assessment for all time periods is listed in Table 3.

**Figure 7:**
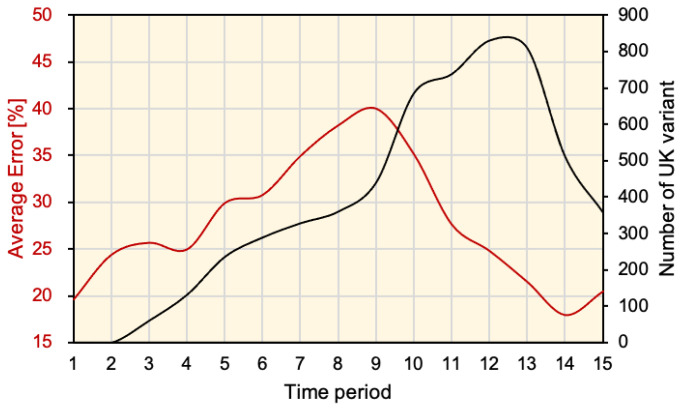
Average relative error in DPC estimation using deep learning in different time periods and the number of new UK viral variants reported in regions of the study.

A comparison between the reported new viral strains and the error of deep learning estimation is shown in Figure 7. The summation number of viral variants in the studied regions reached a peak around mid-April and then decayed. During the spread period, the deep learning forecasting error monotonically increased, which demonstrated the estimation error caused by a new factor that was not included in the training data. From Figure 6, it is clear that the error presented an underestimate of DPC in most cases. In early April, the deep learning forecasting error started to decay. This can be considered as the training data starting to include periods where excessive DPC were reported, and therefore the adaptation and correction were evolving.

### 3.3. Effective Reproduction Number

**Figure 8:**
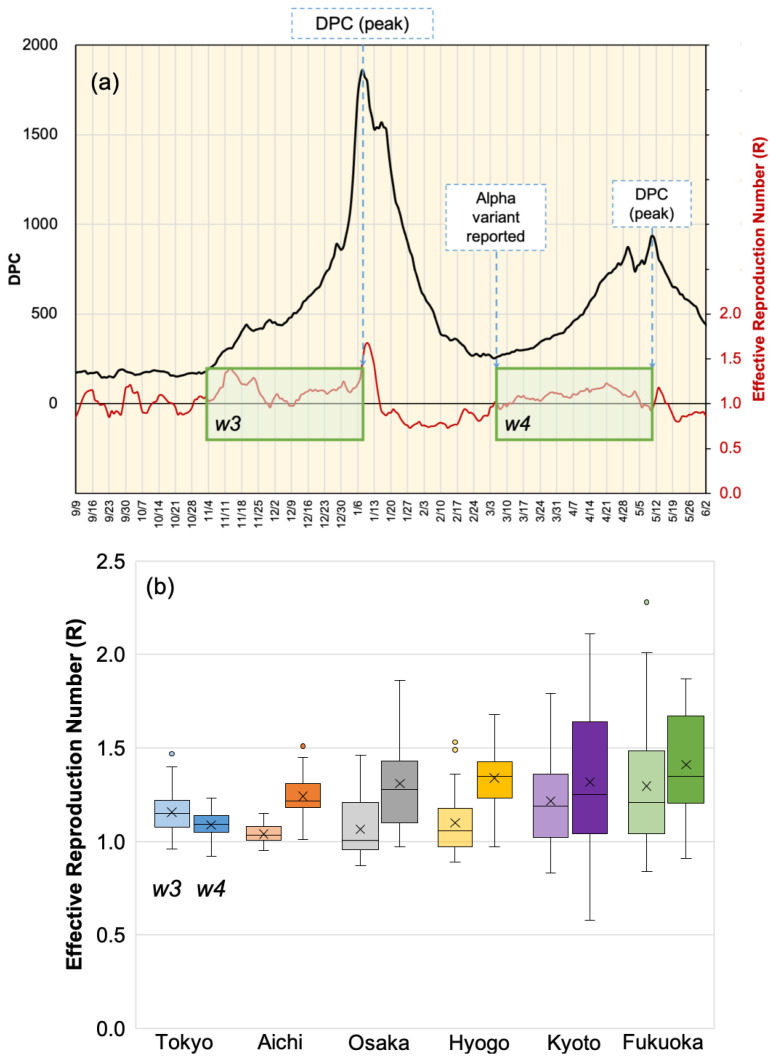
(**a**) Definition of two time slots w3 and w4 preceding the third and fourth pandemic waves, respectively. Time slots ended with the day of the DPC local maximum value and the width was defined by the first day where the Alpha variant was reported. (**b**) Box plot of *R* values computed in w3 (**left**) and w4 (**right**) in different districts.

An important factor in measuring the pandemic spread is the effective reproduction number (*R*). The *R* value computed earlier to the peak of the third wave and fourth wave may demonstrate the viral spread pattern. We defined two identical time slots at each end with the day at which a maximum DPC was reported. The time slot proceeding the fourth wave was defined by the day at which the Alpha variant cases were recognized and reported. The selection of time slots w3 and w4 is shown in Figure 8a. A box plot of the *R* values in different districts are shown in Figure 8b. It is clear that the average *R* values were generally increased in time slots in which the Alpha variant was reported (w4), except for the case of Tokyo. The average *R* value was reduced by 5.8% in Tokyo and increased by 18.9%, 20.26%, 19.23%, 6.00%, and 8.18% in Aichi, Osaka, Hyogo, Kyoto, and Fukuoka, respectively.

**Figure 9:**
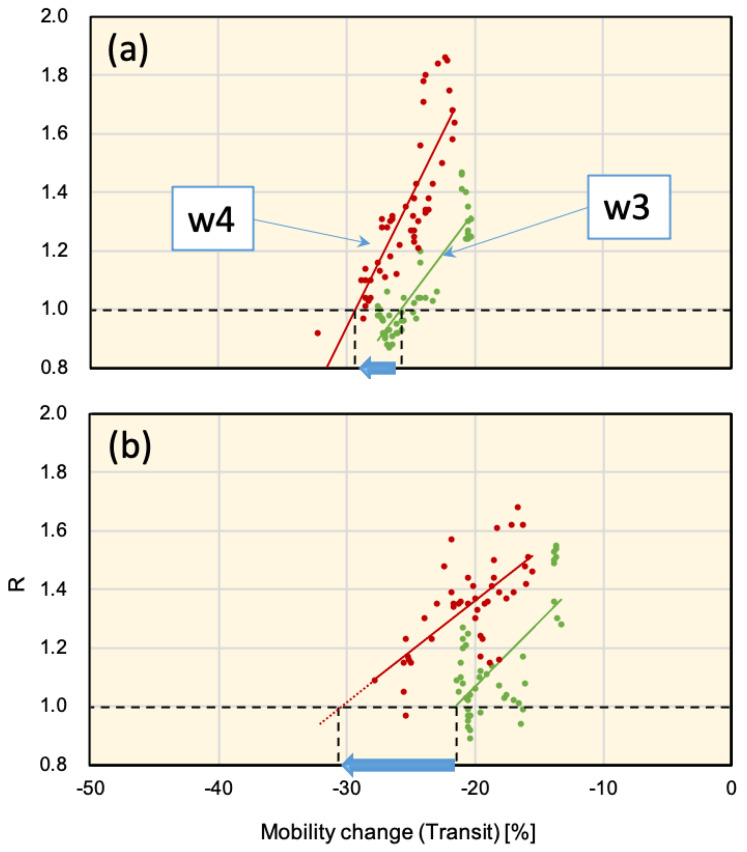
Plots of effective reproduction number (*R*) and corresponding mobility change (Transit) in (**a**) Osaka and (**b**) Hyogo for time slots w3 and w4 defined in Figure 8a. Considering all other factors unchanged, to reduce the upsurge (i.e., reach to *R*=1.0), mobility at transit spots is required to be reduced by 4 and 9 points in Osaka and Hyogo, respectively.

The effect of mobility was a dominant factor in the viral spread as a surrogate for the degree of social distancing. It is important to define a threshold for the mobility change value that reduces the effect of a new viral variant. Moreover, it is also important to consider the effect of the incubation period [36]. Considering a 7-day average of mobility data with 3-day stride, we compute the effective mobility values and study the correlation with *R* values. In Figure 9, a plot of the mobility change percentage and *R* values within the time slots w3 and w4 in Osaka and Hyogo are shown. From this figure it can be concluded that mobility in transit spots needed to be reduced by 4 and 9 points in Osaka and Hyogo, respectively, to compensate the *R* value at the level of 1.0. Moreover, within mobility values −20% to −30%, the *R* values are increased by 22% to 32% in Osaka and Hyogo. A similar conclusion can be drawn for other study area districts and mobility spots and can be a useful reference for SoE enforcement criteria.

## 4. Discussion

An additional burden was discovered with the reports of new SARS-CoV-2 variants. With new mutations, the validity of vaccination and the mortality risk became under question again. The recent sudden increase in infection rates in India shone a light on how the new viral variants could have a strong influence on infection rates [37]. As deep learning becomes a state-of-the-art approach to forecasting COVID-19, we became curious on how this new variable would influence the forecasting accuracy of deep learning models. In many cases, it is difficult to clearly understand and evaluate the contribution of different factors to the quality of the model output due to the “black box” nature of most deep learning models.

We studied a recently developed deep learning model that is proved to be of superior quality [35]. While the network architecture is almost the same as the one in [35], several changes have been considered regarding the data used in training. (1) The mobility data is extended to cover six different zones (retail, grocery, parks, transit, work, and residential) based on Google mobility reports, while only transit mobility was considered in an earlier study. (2) Training data are normalized within each district such that the network can be trained using all study regions in one shot as shown in Figure 4. (3) Additional data consider the workday status and state of emergency calls. We consider several training and testing scenarios over a long time frame to study the effect of new viral variants (specifically, the Alpha mutation). Results of different districts demonstrate interesting features. In general, with the emerging of a new variant, a recognized underestimate of DPC is recognized in all the studied districts which indicate an unexpected infections upsurge. The estimated upsurge in Japan is around 20–40% in DPC and up to 20% in terms of effective reproduction number, which is relatively smaller than those reported in the UK [20]. Later on, when the training data overlaps time frames where variants are reported, forecasting accuracy improves gradually, which demonstrates the network adaptation to the change caused by viral variants. An approximation of four weeks is required for the deep learning model to handle the upsurge caused by the Alpha viral variant. This period sounds reasonable considering the virus incubation time and delay in process of testing and confirmation [38,39].

By considering the number of new viral variants reported within a specific time period, we can clearly understand why the deep learning estimation worked well in some cases and failed in others. The cases of Osaka and Hyogo (neighboring districts in the Kansai region) are similar with a significant number of new viral strains reported compared to all other regions in Japan (Figure 3). Even in the Kansai region, Kyoto was where a small number of viral variants were reported in early March with no subsequent spread. Therefore, the DPC demonstrated slightly high values but still within the estimated range. The data of Aichi demonstrated a case where the viral variants were being reported with an approximate one month delay for similar cases in Osaka and Hyogo. Therefore, the situation was almost normal before mid-April and started to reach values above normal later. The viral variants in Tokyo demonstrated a similar pattern to those in Aichi however, with such a large population in Tokyo, the effect can be milder. Although Aichi is located close to the Kansai area, the new viral variant is not reported simultaneously. Government calls for SoE and announcements from local authorities has a notable impact on public response and can be confirmed by a mobility change during SoE, which generally advises the public to voluntarily reduce incidences that may increase social interaction and potential infection. Moreover, it is likely that the third SoE announced in Tokyo on 25 April 2021 helped to reduce the spread of new viral variants.

**Figure 10:**
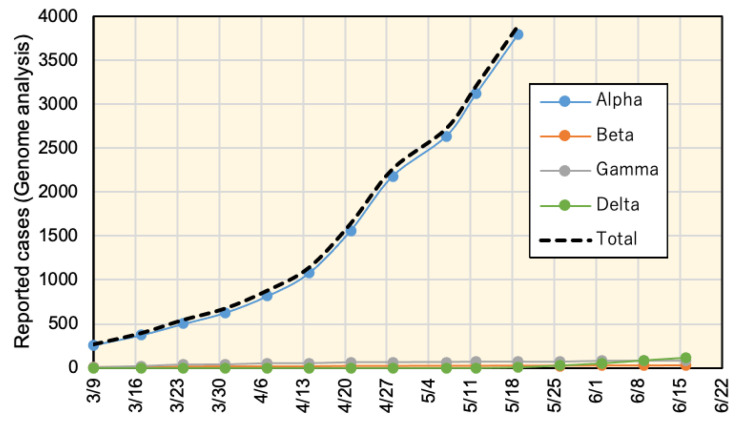

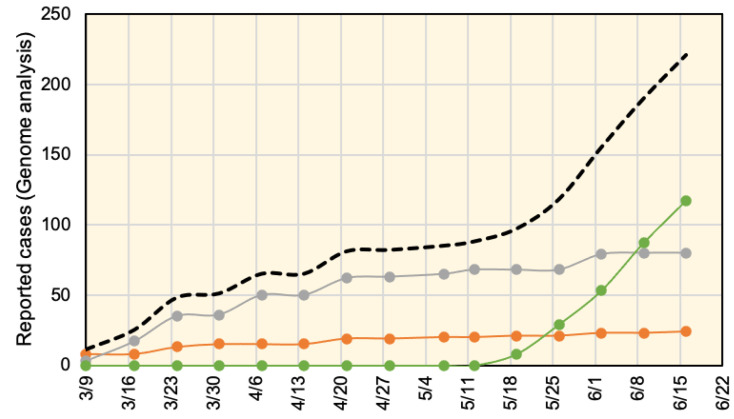
Cumulative number of COVID-19 viral variant reported cases in Japan using genome analysis. The top side of the chart demonstrates all variants, in which the Alpha variant represents approximately 95% of the reported cases. After May 26, the Alpha variant was considered dominant and excluded from the analysis report. The bottom side of the chart shows viral variants excluding Alpha.

Monitoring the status of different viral variants may provide useful insight on the viral spread based on the analysis discussed here. Figure 10 illustrates the reported cases with different variants since early March 2020. Most of the cases (approximately 95%) were the Alpha variant. As per 26 May 2021, this variant has been considered dominant and was excluded from the follow-up reports. The Delta variant started to be recognized on 18 May 2021 and as of the latest report released on 16 June 2021, it was the major variant at 53%. A recent study from Scotland indicated that the Delta virus variant may double the risk for hospitalization compared to the Alpha variant [40]. This would raise alerts for potential expected risk in the near future considering the current pattern of viral variant spreading in Japan.

## 5. Conclusions

We investigated the problem of COVID-19 DPC forecasting with the emergence of new viral variants. This was considered using data from six different districts in Japan and a deep learning model was used to forecast future potential infection cases using meteorological parameters and mobility data. This process was repeated for 15 time-frames with the stride of one week to record changes in forecasting accuracy. Results demonstrated a recognized underestimation in forecasting within the time frames with high viral variant records. Later on, when network training data included time periods in which viral variants were reported, network forecasting accuracy improved gradually. This may indicate that infection rates are increased with the emergence of new viral variants (20–40%), which could not be recognized in a deep learning model trained using earlier data.

## Figures and Tables

**Table 1:** Dataset splitting for training and testing in different forecasting time periods.

Period#	Training		Testing	
	from	to	from	to
1	15 February 2020	26 January 2021	27 January 2021	23 February 2021
2	⋯	2 February 2021	3 February 2021	2 March 2021
3	⋯	9 February 2021	10 February 2021	9 March 2021
4	⋯	16 February 2021	17 February 2021	16 March 2021
5	⋯	23 February 2021	24 February 2021	23 March 2021
6	⋯	2 March 2021	3 March 2021	30 March 2021
7	⋯	9 March 2021	10 March 2021	6 April 2021
8	⋯	16 March 2021	17 March 2021	13 April 2021
9	⋯	23 March 2021	24 March 2021	20 April 2021
10	⋯	30 March 2021	31 March 2021	27 April 2021
11	⋯	6 April 2021	7 April 2021	4 May 2021
12	⋯	13 April 2021	14 April 2021	11 May 2021
13	⋯	20 April 2021	21 April 2021	18 May 2021
14	⋯	27 April 2021	28 April 2021	25 May 2021
15	...	4 May 2021	5 May 2021	1 June 2021

**Table 2:** Average absolute error [%] computed using different scenarios for data blend for all study districts.

Scenarios	1	2	3	4
**Day labels**	✓	✓	✓	✓
**Meteorological data**	✓	✗	✗	✓
**Mobility**	✗	Transit only	✓	✓
**DPC**	✓	✓	✓	✓
Tokyo	46.0	37.3	19.0	16.3
Aichi	26.5	22.6	23.2	23.9
Osaka	26.0	20.9	24.9	18.4
Hyogo	25.1	17.4	12.6	19.5
Kyoto	23.4	24.6	22.0	15.0
Fukuoka	25.6	24.5	27.7	30.7
Average	28.8	24.6	21.6	20.6

**Table 3:** Average absolute error [%] for DPC estimated in different regions and time periods.

Period#	Tokyo	Aichi	Osaka	Hyogo	Kyoto	Fukuoka	Avg.
1	22.66	15.80	21.72	12.87	18.45	26.09	19.60
2	29.40	25.14	19.33	26.64	25.16	20.61	24.38
3	22.75	21.74	23.15	24.34	40.61	21.34	25.66
4	9.35	20.47	25.33	22.99	56.25	15.35	24.96
5	11.31	11.88	24.76	39.81	59.08	32.62	29.91
6	19.75	15.91	32.37	32.53	40.59	43.53	30.78
7	24.20	21.75	42.51	38.40	38.26	44.61	34.95
8	15.73	23.80	49.90	33.02	30.02	76.87	38.22
9	17.15	27.16	52.69	43.10	44.51	55.30	39.99
10	14.68	26.37	38.93	39.97	40.20	50.39	35.09
11	21.72	23.43	24.68	28.21	24.81	42.67	27.59
12	18.16	30.17	18.87	21.54	16.56	43.28	24.77
13	23.99	33.94	7.56	13.82	13.24	36.13	21.45
14	13.80	21.37	23.09	20.29	11.81	17.19	17.93
15	9.37	21.42	24.42	22.50	18.48	26.59	20.46
Avg.	18.27	22.69	28.62	28.00	31.87	36.84	

## Data Availability

The datasets and/or software generated during the current study are available from the corresponding author on reasonable request.

## References

[1] Ozili P.K., Arun T. (2020). Spillover of COVID-19: Impact on the Global Economy. SSRN Electron. J..

[2] De-Leon H., Calderon-Margalit R., Pederiva F., Ashkenazy Y., Gazit D. (2021). First indication of the effect of COVID-19 vaccinations on the course of the outbreak in Israel. medRxiv.

[3] Mofijur M., Fattah I.R., Alam M.A., Islam A.S., Ong H.C., Rahman S.A., Najafi G., Ahmed S., Uddin M.A., Mahlia T. (2021). Impact of COVID-19 on the social, economic, environmental and energy domains: Lessons learnt from a global pandemic. Sustain. Prod. Consum..

[4] Musa S.S., Qureshi S., Zhao S., Yusuf A., Mustapha U.T., He D. (2021). Mathematical modeling of COVID-19 epidemic with effect of awareness programs. Infect. Dis. Model..

[5] Banik R., Rahman M., Sikder T., Gozal D. (2020). COVID-19 in Bangladesh: Public awareness and insufficient health facilities remain key challenges. Public Health.

[6] Sun C.X., He B., Mu D., Li P.L., Zhao H.T., Li Z.L., Zhang M.L., Feng L.Z., Zheng J.D., Cheng Y. (2020). Public Awareness and Mask Usage during the COVID-19 Epidemic: A Survey by China CDC New Media. Biomed. Environ. Sci..

[7] Weiss H.H. (2013). The SIR model and the foundations of public health. Mater. Mat..

[8] Klepac P., Pomeroy L.W., Bjørnstad O.N., Kuiken T., Osterhaus A.D., Rijks J.M. (2009). Stage-structured transmission of phocine distemper virus in the Dutch 2002 outbreak. Proc. R. Soc. B Biol. Sci..

[9] Arik S.O., Li C.L., Yoon J., Sinha R., Epshteyn A., Le L.T., Menon V., Singh S., Zhang L., Yoder N. (2020). Interpretable Sequence Learning for COVID-19 Forecasting. arXiv.

[10] Carli R., Cavone G., Epicoco N., Scarabaggio P., Dotoli M. (2020). Model predictive control to mitigate the COVID-19 outbreak in a multi-region scenario. Annu. Rev. Control..

[11] Rahimi I., Chen F., Gandomi A.H. (2021). A review on COVID-19 forecasting models. Neural Comput. Appl..

[12] Tomar A., Gupta N. (2020). Prediction for the spread of COVID-19 in India and effectiveness of preventive measures. Sci. Total Environ..

[13] Zhang G., Liu X. (2021). Prediction and control of COVID-19 spreading based on a hybrid intelligent model. PLoS ONE.

[14] Noh J., Danuser G. (2021). Estimation of the fraction of COVID-19 infected people in U.S. states and countries worldwide. PLoS ONE.

[15] Devaraj J., Madurai Elavarasan R., Pugazhendhi R., Shafiullah G., Ganesan S., Jeysree A.K., Khan I.A., Hossain E. (2021). Forecasting of COVID-19 cases using deep learning models: Is it reliable and practically significant?. Results Phys..

[16] Mousavi M., Salgotra R., Holloway D., Gandomi A.H. (2020). COVID-19 Time Series Forecast Using Transmission Rate and Meteorological Parameters as Features. IEEE Comput. Intell. Mag..

[17] Balli S. (2021). Data analysis of Covid-19 pandemic and short-term cumulative case forecasting using machine learning time series methods. Chaos Solitons Fractals.

[18] Melin P., Sánchez D., Monica J.C., Castillo O. (2021). Optimization using the firefly algorithm of ensemble neural networks with type-2 fuzzy integration for COVID-19 time series prediction. Soft Comput..

[19] Krutikov M., Hayward A., Shallcross L. (2021). Spread of a Variant SARS-CoV-2 in Long-Term Care Facilities in England. N. Engl. J. Med..

[20] Davies N.G., Abbott S., Barnard R.C., Jarvis C.I., Kucharski A.J., Munday J.D., Pearson C.A., Russell T.W., Tully D.C., Washburne A.D. (2021). Estimated transmissibility and impact of SARS-CoV-2 lineage B.1.1.7 in England. Science.

[21] Volz E., Mishra S., Chand M., Barrett J.C., Johnson R., Geidelberg L., Hinsley W.R., Laydon D.J., Dabrera G., O’Toole Á. (2021). Transmission of SARS-CoV-2 Lineage B.1.1.7 in England: Insights from linking epidemiological and genetic data. medRxiv.

[22] Nouvellet P., Bhatia S., Cori A., Ainslie K.E., Baguelin M., Bhatt S., Boonyasiri A., Brazeau N.F., Cattarino L., Cooper L.V. (2021). Reduction in mobility and COVID-19 transmission. Nat. Commun..

[23] Badr H.S., Du H., Marshall M., Dong E., Squire M.M., Gardner L.M. (2020). Association between mobility patterns and COVID-19 transmission in the USA: A mathematical modelling study. Lancet Infect. Dis..

[24] Kraemer M.U., Yang C.H., Gutierrez B., Wu C.H., Klein B., Pigott D.M., Du Plessis L., Faria N.R., Li R., Hanage W.P. (2020). The effect of human mobility and control measures on the COVID-19 epidemic in China. Science.

[25] Cartenì A., Di Francesco L., Martino M. (2020). How mobility habits influenced the spread of the COVID-19 pandemic: Results from the Italian case study. Sci. Total Environ..

[26] Ma Y., Zhao Y., Liu J., He X., Wang B., Fu S., Yan J., Niu J., Zhou J., Luo B. (2020). Effects of temperature variation and humidity on the death of COVID-19 in Wuhan, China. Sci. Total Environ..

[27] Xie J., Zhu Y. (2020). Association between ambient temperature and COVID-19 infection in 122 cities from China. Sci. Total Environ..

[28] Wu Y., Jing W., Liu J., Ma Q., Yuan J., Wang Y., Du M., Liu M. (2020). Effects of temperature and humidity on the daily new cases and new deaths of COVID-19 in 166 countries. Sci. Total Environ..

[29] Rashed E.A., Kodera S., Gomez-Tames J., Hirata A. (2020). Influence of Absolute Humidity, Temperature and Population Density on COVID-19 Spread and Decay Durations: Multi-Prefecture Study in Japan. Int. J. Environ. Res. Public Health.

[30] Kodera S., Rashed E.A., Hirata A. (2020). Correlation between COVID-19 Morbidity and Mortality Rates in Japan and Local Population Density, Temperature, and Absolute Humidity. Int. J. Environ. Res. Public Health.

[31] Diao Y., Kodera S., Anzai D., Gomez-Tames J., Rashed E.A., Hirata A. (2021). Influence of population density, temperature, and absolute humidity on spread and decay durations of COVID-19: A comparative study of scenarios in China, England, Germany, and Japan. One Health.

[32] Majumder P., Ray P.P. (2021). A systematic review and meta-analysis on correlation of weather with COVID-19. Sci. Rep..

[33] Briz-Redón Á., Serrano-Aroca Á. (2020). The effect of climate on the spread of the COVID-19 pandemic: A review of findings, and statistical and modelling techniques. Prog. Phys. Geogr. Earth Environ..

[34] Espejo W., Celis J.E., Chiang G., Bahamonde P. (2020). Environment and COVID-19: Pollutants, impacts, dissemination, management and recommendations for facing future epidemic threats. Sci. Total Environ..

[35] Rashed E.A., Hirata A. (2021). One-year lesson: Machine learning prediction of COVID-19 positive cases with meteorological data and mobility estimate in Japan. Int. J. Environ. Res. Public Health.

[36] Lauer S.A., Grantz K.H., Bi Q., Jones F.K., Zheng Q. (2020). The Incubation Period of Coronavirus Disease 2019 (COVID-19) From Publicly Reported Confirmed Cases: Estimation and Application. Ann. Intern. Med..

[37] Thiagarajan K. (2021). Why is India having a COVID-19 surge?. BMJ.

[38] Zaki N., Mohamed E.A. (2021). The estimations of the COVID-19 incubation period: A scoping reviews of the literature. J. Infect. Public Health.

[39] Omori R., Mizumoto K., Chowell G. (2020). Changes in testing rates could mask the novel coronavirus disease (COVID-19) growth rate. Int. J. Infect. Dis..

[40] Sheikh A., McMenamin J., Taylor B., Robertson C. (2021). SARS-CoV-2 Delta VOC in Scotland: Demographics, risk of hospital admission, and vaccine effectiveness. Lancet.

